# Link Prediction in Weighted Networks: A Weighted Mutual Information Model

**DOI:** 10.1371/journal.pone.0148265

**Published:** 2016-02-05

**Authors:** Boyao Zhu, Yongxiang Xia

**Affiliations:** College of Information Science and Electronic Engineering, Zhejiang University, Hangzhou 310027, China; Universidad Rey Juan Carlos, SPAIN

## Abstract

The link-prediction problem is an open issue in data mining and knowledge discovery, which attracts researchers from disparate scientific communities. A wealth of methods have been proposed to deal with this problem. Among these approaches, most are applied in unweighted networks, with only a few taking the weights of links into consideration. In this paper, we present a weighted model for undirected and weighted networks based on the mutual information of local network structures, where link weights are applied to further enhance the distinguishable extent of candidate links. Empirical experiments are conducted on four weighted networks, and results show that the proposed method can provide more accurate predictions than not only traditional unweighted indices but also typical weighted indices. Furthermore, some in-depth discussions on the effects of weak ties in link prediction as well as the potential to predict link weights are also given. This work may shed light on the design of algorithms for link prediction in weighted networks.

## Introduction

The problem of link prediction attempts to uncover missing links and predict the emergence of future links in complex networks based on the available information, such as observed links and nodes’ attributes [1–3]. Because of its broad applications in various domains, the study of link prediction has become a research hotspot. In some biological networks, such as protein-protein interaction networks and metabolic networks [4, 5], the discovery of interaction links is usually expensive. Therefore, accurate predictors can be applied for one to seek the most promising latent links, which will cost less than blindly checking all possible interaction connections [6, 7]. With the overload of information nowadays, the dependence of people on information filtering systems, such as recommender systems, is increasing [8, 9]. In this sense, link prediction can serve as a significant technique in recommender systems, such as e-commerce recommendation [10] and friendship recommendation [11, 12]. Moreover, the technique of link prediction has been successfully applied to evaluate network evolving models [13, 14], and also to identify spurious links [6]. Recently, the link-predictability problem was proposed to characterize the extent of links in a network could be predicted [15]. Accordingly, this can help us understand the organization of real networks.

Plenty of link prediction methods based on network structures have been proposed in the past years [16–20]. Among various approaches, *Common Neighbors* (CN) is the simplest one, which assumes that two nodes are more likely to form a link if they have more common neighbors. However, CN simply counts the number of common neighbors but ignores their different contributions on the connection likelihood. Hence, many variants of CN have been put forward to further boost the prediction accuracy by improving the discriminative extent of candidate links, such as *Adamic-Adar* (AA) [16] and *Resource Allocation* (RA) [17], where a common neighbor with low degree is advocated via assigning more weight on it. Based on the Bayesian theory, a local naïve Bayes model [18] was presented to differentiate the role of different common neighbors. In addition, node centrality (degree, closeness and betweenness) was also applied to make common neighbors more distinguishable [19]. Recently, Tan *et al*. [20] reexamined the role of common neighbors from the perspective of information theory, and the contributions of common neighbors are differentiated by the mutual information of local structures.

Most of previous studies on link prediction focused on unweighted networks but ignored the naturally existed link weights. Up to now, little literature is available on link prediction in weighted networks. Murata and Moriyasu [21] proposed the variants of CN, AA and RA as weighted indices for predicting the emergence of communications between users in social networks. It was revealed that proximities between nodes can be estimated better by using both graph proximity measures and the weights of existing links. In some networks, especially in social networks, weak ties may play a more important role than strong ties [22, 23]. Lü and Zhou [24] investigated the role of weak ties in link prediction and suggested that emphasis on the contributions of weak ties can remarkably enhance the prediction accuracy. Sá and Prudêncio [25] studied the relevance of using link weights to improve supervised link prediction. Results proved that the prediction accuracy could be improved by using weights on the links.

In this paper, a weighted mutual information model is developed by gaining the benefits from both structural properties and link weights. In our model, the mutual information is adopted to estimate the effect of network structures on the connection likelihood. Different from the estimation of mutual information in Ref [20], we employ a more rigorous theoretic way here. Besides, the weights of links are applied to further emphasize the discriminative resolution of candidate links. Empirical experiments on four real-world weighted networks reveal that the proposed method improves the prediction accuracy substantially compared with not only traditional unweighted indices but also typical weighted indices. In addition, we also give some in-depth discussions on the role of weak ties in link prediction as well as the potential to predict link weights. We hope this work will provide some inspirations about how to incorporate the weights for link prediction in weighted networks.

## Materials and Methods

### Data and Problem Description

Four weighted networks from disparate fields are considered in our experiments. 1) Celegans: the neural network of the nematode worm *C. elegans*, where a node stands for a neuron, a link joins two neurons if they have synaptic contacts, and the weight represents the number of synapses between two neurons [26]. This network has 297 neurons and 2148 synaptic contacts. 2) USAir: the network of US air transportation, where the weight of a link is the frequency of flights between two airports [27]. This network contains 332 airports and 2126 airlines. 3) Baywet: the network which contains the carbon exchanges in the cypress wetlands of south Florida during the wet season [28], where a node represents a taxon, and an edge denotes that a taxon uses another taxon as food with a given trophic factor (feeding level). This network has 123 nodes and 2106 edges. 4) Bible: the lexical network with the nouns in King James Bible and information about their occurrences [28], where a node stands for a noun and a link indicates that two nouns appear together in the same verse. The weight on a link represents how often two nouns occurred together. This network contains 1773 nodes and 9131 edges.

In this paper, only an undirected weighted network *G*(*V*, *E*, *W*) is studied, where *V*, *E* and *W* denote sets of nodes, links and link weights, respectively. Note that, *W*_*xy*_ = *W*_*yx*_, where *W*_*xy*_ stands for the weight on link (*x*, *y*). Multiple links and self-loops are not allowed here. The task of link prediction is to discover missing links or predict future links. To do this, for each non-existent node pair, namely a link (*x*, *y*) ∈ *U* − *E*, where *U* stands for the universal set, we assign a score *s*_*xy*_ to quantify the connection likelihood of nodes *x* and *y*. A higher score means higher probability that nodes *x* and *y* will form a link. All the non-existent links are sorted by their scores in descending order, and the links with highest ranks are most likely to appear.

To validate the prediction performance of a predictor, the observed links, *E*, are randomly divided into two parts: training set *E*^*T*^, is regarded as given information, and probe set *E*^*P*^, is only used for testing. Clearly, we have *E*^*T*^ ∪ *E*^*P*^ = *E* and E^*T*^ ∩ E^*P*^ = ⌀. In this paper, the training set always contains 90% of observed links, and the rest constitutes the probe set. We apply a standard metric called *Precision* to quantify the accuracy of prediction, which is defined as the ratio of true missing links in the predicted link set, *i.e*., if top *L* links are treated as predicted links while *L*_*r*_ of which are in the probe set, then the value of Precision equals to *L*_*r*_/*L*.

### Weighted Similarity Indices Based on Local Information

In most real-world networks, links are naturally weighted. The weight of a link may represent different meanings in different networks, such as the number of synapses and gap junctions in neural networks, the carbon flow between species in food webs or the amount of traffic load along connections in transportation networks. Murata and Moriyasu [21] studied the way to extend similarity indices from unweighted networks to weighted networks. Based on this method, the weighted cases of CN, AA and RA (named as WCN, WAA and WRA, respectively) are defined as [21, 24]

$$\begin{array}{c} s_{xy}^{WCN}=\sum_{z\in O_{xy}}\left(W_{xz}+W_{zy}\right), \end{array}$$ (1)

$$\begin{array}{c} s_{xy}^{WAA}=\sum_{z\in O_{xy}}\frac{W_{xz}+W_{zy}}{\log(1+S_{z})}, \end{array}$$ (2)

$$\begin{array}{c} s_{xy}^{WRA}=\sum_{z\in O_{xy}}\frac{W_{xz}+W_{zy}}{S_{z}}, \end{array}$$ (3)

where *O*_*xy*_ represents the common neighbor set of node pair (*x*, *y*), which can be written as *O*_*xy*_ = {*z* : *z* ∈ Γ(*x*) ∩ Γ(*y*)}. Γ(*x*) stands for the set of neighbors of node *x*. *W*_*xz*_ is the weight of link (*x*, *z*). *S*_*z*_ denotes the strength of node *z*, *i.e*., the sum of weights of links directly connected with node *z*, which is defined as *S*_*z*_ = ∑_*z*′∈Γ(*z*)_ *W*_*zz*′_.

For some networks, weak ties may play a more important role than strong ties in link prediction [24]. In order to investigate the role of weak ties in predicting missing links, Lü and Zhou [24] introduced a free parameter, *α*, to control the relative contributions of weak ties to the similarity measures. The indices WCN, WAA and WRA with parameters (denoted as WCN_*α*_, WAA_*α*_ and WRA_*α*_, respectively) are

$$\begin{array}{c} s_{xy}^{WCN_{\alpha }}=\sum_{z\in O_{xy}}\left(W_{xz}^{\alpha }+W_{zy}^{\alpha }\right), \end{array}$$ (4)

$$\begin{array}{c} s_{xy}^{WAA_{\alpha }}=\sum_{z\in O_{xy}}\frac{W_{xz}^{\alpha }+W_{zy}^{\alpha }}{\log(1+S_{z})}, \end{array}$$ (5)

$$\begin{array}{c} s_{xy}^{WRA_{\alpha }}=\sum_{z\in O_{xy}}\frac{W_{xz}^{\alpha }+W_{zy}^{\alpha }}{S_{z}}, \end{array}$$ (6)

where $S_{z}=\sum_{z^{\prime }\in \Gamma \left(z\right)}W_{zz^{\prime }}^{\alpha }$. Note that, when *α* = 0, *S*_*z*_ is the degree of node *x*, and the indices degenerate to the unweighted forms, namely CN, AA and RA. On the other hand, when *α* = 1, the indices are the simply weighted cases, as shown in Eqs (1)–(3).

## Results

### Weighted Mutual Information Model

Considering a pair of disconnected nodes (*x*, *y*), our task is to determine a prediction measure that uses not only the structural properties of common neighbors of this node pair but also weights on corresponding links. As reported in literature [18, 19], different common neighbors may have different contributions on the connection likelihood. Here we investigate the role of common neighbors from the perspective of mutual information [20, 29–32]. First of all, for the sake of brevity, some definitions about self-information and mutual information are given, respectively.

For two events (or random variables) *X* and *Y*, the conditional probability mass function is *p*(*x*|*y*) (*x* ∈ *X*, *y* ∈ *Y*), and the marginal probability mass functions are *p*(*x*) and *p*(*y*), respectively. The mutual information of two outcomes *x*_*i*_ and *y*_*j*_ (*x*_*i*_ ∈ *X*, *y*_*j*_ ∈ *Y*) can be derived as

$$\begin{array}{ccc} I(x_{i};y_{j}) & = & \log\frac{p(x_{i}|y_{j})}{p(x_{i})}\\ & = & -\log p\left(x_{i}\right)-\left(-\log p\left(x_{i}|y_{j}\right)\right)\\ & = & I\left(x_{i}\right)-I\left(x_{i}|y_{j}\right), \end{array}$$ (7)

where *I*(*x*_*i*_|*y*_*j*_) is the conditional self-information, which indicates the uncertainty of the occurrence of outcome *x*_*i*_ given that outcome *y*_*j*_ happens, and *I*(*x*_*i*_) is the self information that quantifies the uncertainty of outcome *x*_*i*_.

The mutual information measures how much the uncertainty about one event can be reduced by giving the outcome of the other event. Therefore, if two events are independent from each other, the mutual information equals to zero.

Now consider the link-prediction problem. From the perspective of information theory, the estimation of connection likelihood between a pair of nodes can be treated as calculating the information of the event that two nodes are connected. More specifically, for a non-connected node pair (*x*, *y*), we use $L_{xy}^{1}$ to denote the event that nodes *x* and *y* are connected. If the common neighbor set *O*_*xy*_ is available, then the link likelihood can be estimated by $-I(L_{xy}^{1}|O_{xy})$ [20, 32]. According to the definitions of information, $I(L_{xy}^{1}|O_{xy})$ can be written as

$$\begin{array}{c} I\left(L_{xy}^{1}|O_{xy}\right)=I\left(L_{xy}^{1}\right)-I\left(L_{xy}^{1};O_{xy}\right), \end{array}$$ (8)

where $I(L_{xy}^{1};O_{xy})$ is the mutual information between the event that node pair (*x*, *y*) has one link and the event that node pair’s common neighbors are given. $I(L_{xy}^{1})$ can be calculated through the prior probability

$$\begin{array}{c} p\left(L_{xy}^{1}\right)=\frac{M^{T}}{M}, \end{array}$$ (9)

where *M*^*T*^ = |*E*^*T*^| and $M=\frac{\left|V|(|V|-1\right)}{2}$. | ⋅ | denotes the cardinality of the set. Since the prior probabilities $p(L_{xy}^{1})$ are the same for every pair of nodes, here we define the connection likelihood as

$$\begin{array}{c} s_{xy}=I\left(L_{xy}^{1};O_{xy}\right). \end{array}$$ (10)

If the elements of *O*_*xy*_ are supposed to be independent from each other, then

$$\begin{array}{c} I\left(L_{xy}^{1};O_{xy}\right)=\sum_{z\in O_{xy}}I(L_{xy}^{1};z). \end{array}$$ (11)

Instead of estimating $I(L_{xy}^{1};z)$ by averaging the mutual information over all node pairs connected to node *z* as presented in Ref [20], according to the definition Eq (7), $I(L_{xy}^{1};z)$ can be calculated more accurately through

$$\begin{array}{c} I\left(L_{xy}^{1};z\right)=I\left(L_{xy}^{1}\right)-I\left(L_{xy}^{1}|z\right), \end{array}$$ (12)

where $I(L_{xy}^{1}|z)$ is the conditional self-information of the event that node pair (*x*, *y*) have one link given that their common neighbor *z* is available. To calculate $I(L_{xy}^{1}|z)$, we need to obtain $p(L_{xy}^{1}|z)$. Generally speaking, $p(L_{xy}^{1}|z)$ can be estimated by the clustering coefficient of node *z*, *C*_*z*_, which is defined as

$$\begin{array}{c} p\left(L_{xy}^{1}|z\right)=C_{z}=\frac{N_{▵z}}{N_{▵z}+N_{\wedge z}}, \end{array}$$ (13)

where *N*_△*z*_ and *N*_∧*z*_ are the numbers of connected and disconnected node pairs who share the common neighbor *z*, respectively.

Altogether, we can obtain

$$\begin{array}{ccc} s_{xy} & = & \sum_{z\in O_{xy}}I(L_{xy}^{1};z)\\ & = & \sum_{z\in O_{xy}}\left(I\left(L_{xy}^{1}\right)-I\left(L_{xy}^{1}|z\right)\right)\\ & = & \sum_{z\in O_{xy}}\left(-\log p\left(L_{xy}^{1}\right)+\log p\left(L_{xy}^{1}|z\right)\right)\\ & = & \sum_{z\in O_{xy}}\left(-\log\frac{M^{T}}{M}+\log\frac{N_{▵z}}{N_{▵z}+N_{\wedge z}}\right). \end{array}$$ (14)

Note that, if nodes *x* and *y* do not own any common neighbor, $I(L_{xy}^{1};z)$ equals to zero. Clearly, if *C*_*z*_ = 1 for all nodes, then *s*_*xy*_ degenerates to CN. Therefore, according to the clustering coefficient *C*_*z*_, different common neighbors offer different contributions on the connection likelihood.

Next, we will introduce how to enhance the accuracy of link prediction with link weights. In particular, CN-based unweighted indices have poor performance in low clustering networks [18]. In this case, additional information is needed to break the bottleneck. In WCN, WAA and WRA, the weights of links connecting common neighbors to the corresponding node pair are used to facilitate link prediction. Under this motivation, we add a weight function *f*(*W*_*xz*_, *W*_*zy*_) in Eq (14) to combine the benefits from both structural properties and link weights, and obtain

$$\begin{array}{c} s_{xy}^{WMI}=\sum_{z\in O_{xy}}f\left(W_{xz},W_{zy}\right)I\left(L_{xy}^{1};z\right). \end{array}$$ (15)

The proposed model is called Weighted Mutual Information (WMI). Although the expression of WMI model is similar to that of local naïve Bayes model [18], they are inspired by different motivations. The former is motivated by the combination of the benefits from both structure information and link weights, while the latter focuses on only network structures and tries to drill down the structure information. Here we apply Eqs (1)–(3) as the weight functions, and get the WMI forms of WCN, WAA and WRA, respectively:

$$\begin{array}{c} s_{xy}^{WMI-WCN}=\sum_{z\in O_{xy}}\left(W_{xz}+W_{zy}\right)I\left(L_{xy}^{1};z\right) \end{array}$$ (16)

$$\begin{array}{c} s_{xy}^{WMI-WAA}=\sum_{z\in O_{xy}}\frac{W_{xz}+W_{zy}}{\log(1+S_{z})}I\left(L_{xy}^{1};z\right) \end{array}$$ (17)

$$\begin{array}{c} s_{xy}^{WMI-WRA}=\sum_{z\in O_{xy}}\frac{W_{xz}+W_{zy}}{S_{z}}I\left(L_{xy}^{1};z\right) \end{array}$$ (18)

Besides, in order to emphasize the role of weak ties in link prediction, we define the parameter-dependent versions of Eqs (16)–(18) as follows.

$$\begin{array}{c} s_{xy}^{WMI-WCN_{\alpha }}=\sum_{z\in O_{xy}}\left(W_{xz}^{\alpha }+W_{zy}^{\alpha }\right)I\left(L_{xy}^{1};z\right) \end{array}$$ (19)

$$\begin{array}{c} s_{xy}^{WMI-WAA_{\alpha }}=\sum_{z\in O_{xy}}\frac{W_{xz}^{\alpha }+W_{zy}^{\alpha }}{\log(1+S_{z})}I\left(L_{xy}^{1};z\right) \end{array}$$ (20)

$$\begin{array}{c} s_{xy}^{WMI-WRA_{\alpha }}=\sum_{z\in O_{xy}}\frac{W_{xz}^{\alpha }+W_{zy}^{\alpha }}{S_{z}}I\left(L_{xy}^{1};z\right) \end{array}$$ (21)

In order to distinguish the parameter-dependent versions Eqs (19)–(21) from the non-parameter ones Eqs (16)–(18), we call the latter *pure WMI-based indices* in the following discussions.

### Experimental Results

Table 1 presents the comparison of our WMI model and other several typical unweighted methods under the measure of Precision. As literature [2, 18–20, 24] suggested, the top *L* is set 100 in our experiments. According to the simulation results, without considering the fact of weak ties, the pure WMI-based indices achieve much higher prediction accuracy than the corresponding basic unweighted forms, namely CN, AA and RA, for Celegans and Baywet. In Baywet, the Precision value of WMI-WRA is even improved by nearly 10% compared with RA. In addition, we also give the comparison of our WMI model to the Local Naïve Bayes model (LNB) proposed in paper [18]. LNB-CN, LNB-AA and LNB-RA are the LNB forms of CN, AA and RA, respectively. Compared with the LNB model, pure WMI-based indices provide competitive prediction accuracy in Celegans and Baywet. Especially in Celegans, as the clustering coefficient is low (0.292, the lowest among the four networks), the LNB model can’t improve the discriminative resolution of candidate links [18]; while with the help of weights on corresponding links, the WMI model makes them more distinguishable. Moreover, a comparison with node centrality based method [19] is also given in Table 1. Since the DC-CN index has the best overall performance among the node centrality based approaches, we only compare its optimal version with our model. From the results, except for USAir, our model shows competitive prediction accuracy with the DC-CN index. Further more, if we consider the parameter-dependent versions Eqs (19)–(21) which take the role of weak ties into consideration, the prediction accuracy is enhanced substantially, and our WMI-based indices can achieve the best performance in Celegans, Baywet and Bible.

**Table 1:** Comparison of WMI-based methods with other typical unweighted indices measured by Precision (top-100) on four networks. Each value is obtained by averaging over 100 independent runs of random division of training set and probe set. The abbreviations WMI-WCN*, WMI-WAA* and WMI-WRA* represent highest Precision values obtained by Eqs (19)–(21), respectively. The optimal values of *α* are presented in Table 2. The best performance in each network is marked by bold font.

Indices∖Nets	USAir	Celegans	Baywet	Bible
CN	0.606	0.14	0.092	0.447
LNB-CN	0.621	0.14	0.11	0.539
WMI-WCN	0.498	0.177	0.099	0.398
WMI-WCN*	0.65	**0.198**	0.162	0.55
AA	0.625	0.14	0.093	0.571
LNB-AA	0.641	0.14	0.109	0.747
WMI-WAA	0.549	0.173	0.103	0.466
WMI-WAA*	0.667	0.196	0.164	0.706
RA	0.645	0.133	0.093	0.872
LNB-RA	0.643	0.133	0.107	0.916
WMI-WRA	0.59	0.159	0.192	0.912
WMI-WRA*	0.654	0.165	**0.198**	**0.924**
DC-CN*	**0.668**	0.143	0.094	0.876

From above results, it demonstrates that link weights could be applied to facilitate link prediction. In addition, the fact of weak ties needs to be emphasized in some networks, because weak ties may play a more significant role than strong ties in the prediction [24].

**Fig 1:**
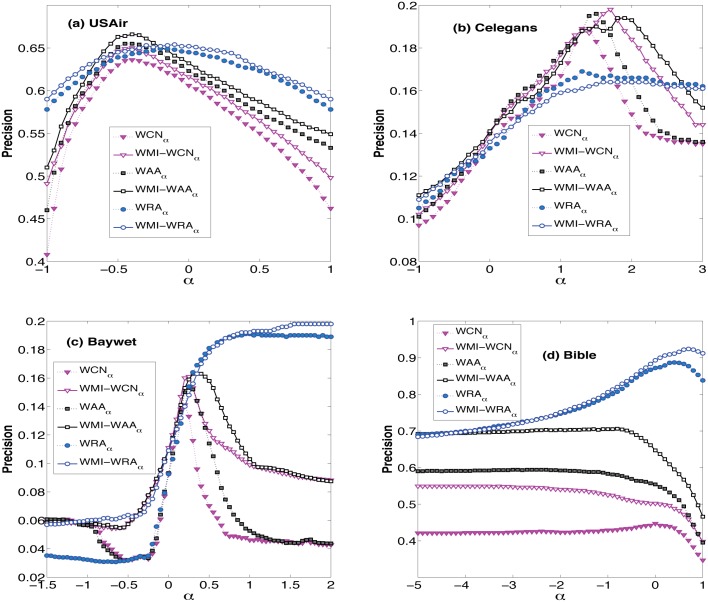
The performances of WMI-based methods and other three weighted indices with different *α* on the four real-world networks.

In order to further explore the role of weak ties in link prediction, the performances of parameter-dependent WMI-based indices with different *α* on four real-world networks are presented in Fig 1. And the optimal values of *α* are given in Table 2. From the results, we can find that the WMI-based indices obtain the best Precision values when *α* is smaller than 1 in USAir, Baywet and Bible, except for WMI-WRA_*α*_ in Baywet. That means the link weights may not show the real strength of ties. Sometimes, the weak ties have a higher strength than their weights suggest. On the other hand, in Celegans, the optimal values of *α* are all greater than 1 for the WMI model, which on the contrary indicates that in some networks the role of weak ties can be as weak as their weights indicate. These results agree with the findings in Ref [24], which used different link prediction indices. This fact reveals that the role of weak ties is an essential characteristic of networks themselves, rather than the detailed link prediction method.

**Table 2:** Optimal values of parameter *α* subject to the highest Precision values in four networks.

Indices∖Nets	USAir	Celegans	Baywet	Bible
WCN*	-0.41	1.41	0.18	0
WMI-WCN*	-0.4	1.71	0.21	-4.16
WAA*	-0.40	1.44	0.25	-2.34
WMI-WAA*	-0.41	1.95	0.32	-0.82
WRA*	-0.24	1.56	0.98	0.4
WMI-WRA*	-0.1	1.76	1.82	0.68

Finally, the performances of our WMI-based indices are compared to other weighted indices given by Eqs (1)–(6) and the reliable-route based methods [33]. As the results shown in Table 3, except for Celegans, all the pure WMI indices achieve better prediction accuracy than corresponding indices (*i.e*., WCN, WAA and WRA). Compared with the reliable-route based methods, namely, rWCN, rWAA and rWRA, the WMI model has better performance in all example networks except for Bible. Since the weighted indices given by Eqs (4)–(6) are parameter-dependent, which consider the role of weak ties as well, the results with different parameter *α* are also shown in Fig 1. From the results, we can conclude that the parameter-dependent WMI-based indices have consistent tendency with their basic weighted forms (*i.e*., WCN_*α*_, WAA_*α*_ and WRA_*α*_) in four real-world networks. In USAir, Baywet and Bible, the WMI model overwhelms its corresponding basic weighted forms almost at any *α* values, especially in USAir. Note that when *α* = 0, the example networks are all turned into unweighted forms, i.e. every link has the same weight. That’s to say, compared with their basic weighted forms, the parameter-dependent WMI-based indices also have a better performance in unweighted networks according to Fig 1. If only consider the optimal results given by Table 3, we can find that except Celegans, the WMI-based indices achieve better prediction accuracy than their counterparts. In Celegans, the WMI-based indices also have nearly the same performance with their counterparts, and WMI-WCN* achieves the best performance among sixteen indices. Altogether, the WMI-based indices overwhelm the compared weighted indices.

**Table 3:** Comparison of WMI-based methods with other typical weighted indices measured by Precision (top-100) on four networks. Each value is obtained by averaging over 100 independent runs of random division of training set and probe set. The abbreviations WCN*, WAA*, WRA*, WMI-WCN*, WMI-WAA* and WMI-WRA* represent the highest Precision values shown in Fig 1 (please refer to detailed *α* values in Table 2). The best performance in each network is marked by bold font.

Indices∖Nets	USAir	Celegans	Baywet	Bible
WCN	0.462	0.167	0.046	0.347
rWCN	0.115	0.133	0.059	0.429
WMI-WCN	0.498	0.177	0.099	0.398
WCN*	0.637	0.189	0.141	0.447
WMI-WCN*	0.65	**0.198**	0.162	0.55
WAA	0.533	0.178	0.053	0.359
rWAA	0.030	0.136	0.067	0.669
WMI-WAA	0.549	0.173	0.103	0.466
WAA*	0.655	0.197	0.153	0.594
WMI-WAA*	**0.667**	0.196	0.164	0.706
WRA	0.578	0.163	0.191	0.838
rWRA	0.134	0.128	0.072	0.817
WMI-WRA	0.59	0.159	0.192	0.912
WRA*	0.647	0.167	0.191	0.887
WMI-WRA*	0.654	0.165	**0.198**	**0.924**

Our experiments are conducted on a desktop computer with 8GB RAM and a Intel (R) Core (TM) i5-3470 CPU @ 3.20 GHz quad-core processor. To illustrate the computing efficiency of each predictor, we summarize their detailed computation time on four real-world networks in Table 4. The results indicate that the WMI based methods overwhelm the DC-CN index, and have relative high computing time but remain similar time scale to other unweighted and weighted methods.

**Table 4:** Comparison of the computing efficiency of sixteen methods on four real-world networks. Each value is the average time in seconds for 100 independent runs.

Indices∖Nets	USAir	Celegans	Baywet	Bible
CN	0.0134	0.0142	0.00484	0.239
WCN	0.0288	0.0297	0.0106	0.502
rWCN	0.043	0.042	0.017	0.75
LNB-CN	0.0606	0.06	0.023	1.08
WMI-WCN	0.0897	0.0902	0.0389	1.53
AA	0.108	0.106	0.0431	1.92
WAA	0.124	0.121	0.0528	2.21
rWAA	0.142	0.139	0.0573	2.59
LNB-AA	0.161	0.156	0.0628	2.91
WMI-WAA	0.191	0.185	0.0748	3.38
RA	0.207	0.201	0.0822	3.76
WRA	0.229	0.22	0.0894	4.15
rWRA	0.246	0.24	0.0925	4.53
LNB-RA	0.265	0.259	0.102	4.85
WMI-WRA	0.295	0.288	0.115	5.31
DC-CN	0.313	0.304	0.121	5.69

In conclusion, the WMI model has better performance over other methods on weights networks and experiences reasonable time complexity.

In practice, the choice of *α* in Eqs (4)–(6) and (19)–(21) still remains a problem. However, as we discussed above, if the strong ties have a significant role than weak ties, it’s a good choice to set the value of *α* as 1 directly. For instance, in Celegans, all those methods perform well when incorporating weights with *α* = 1. Conversely, if the weak ties need to be emphasized, the selection of *α* is usually not easy. A widely applied approach is to divide the training set into two parts, and select one part as the validation set to search for an appropriate *α*. In Table 5, we randomly divide the original network into three parts: training set, validation set and test set, with a proportion 80%, 10% and 10% of the size of original network, respectively, and obtain the estimated optimal *α* values. Then we calculate the RMSD of the Precision values with the estimated and optimal *α*, respectively. From the results, we can find that the differences by applying the estimated values of *α* are small and acceptable, compared to using the optimal *α* in Table 2. Therefore, it’s practical to employ this method to achieve an eligible *α* value.

**Table 5:** Estimated optimal values of parameter *α* subject to the highest Precision values validated by the validation sets in four networks, respectively. The original network is divided into three parts: training set, validation set and probe set. The proportions are 80%, 10% and 10%, respectively. RMSD is the root mean-square deviation of the Precision values with estimated *α* values and optimal *α* values in Table 2, respectively, in each network.

Indices∖Nets	USAir	Celegans	Baywet	Bible
WCN*	-0.07	1.26	0.18	0
WMI-WCN*	-0.21	1.54	0.23	-1.82
WAA*	0	1.44	0.27	-0.79
WMI-WAA*	-0.05	1.18	0.37	-0.99
WRA*	0.41	1.61	2.75	0.48
WMI-WRA*	0.47	1.66	3.34	0.6
RMSD	0.025	0.006	0.002	0.008

### Discussion

According to the empirical experiments, it demonstrates that the weak ties play different roles in different networks. For instance, the role of weak ties is more important than the role of strong ties in USAir, while on the contrary in Celegans. In Ref [24], a motif analysis of example networks is applied to elaborate the role of weak ties in link prediction. Here we try to get an in-depth understanding of the effects of weak ties from a different point of view.

Among the similarity-based methods that incorporate link weights, one latent assumption is that the weights quantify the similarities or affinities between nodes. In other words, larger weights indicate closer relationship between nodes. For example, in Celegans, the weight of a link stands for the number of synapses between a neuron pair. If two neurons have many synaptic contacts, we believe that they have a close relationship with each other. Therefore, the weights describe the similarities between nodes positively. Under this condition, from Eqs (1)–(3) and (16)–(18), it can be concluded that if larger weights are assigned on the links connecting the common neighbors to candidate node pairs, the higher probability of the existence of links can be achieved. In this way, such weights are positively correlated with the connection likelihoods of links. Therefore, the role of weak ties should be depressed, while the role of strong ties, on the contrary, need to be advocated. As a result, the role of weak ties in Celegans is as weak as indicated by the results in Table 2.

However, not all the weights of networks exhibit similarities between nodes. It dependents on the network background. Specifically, the weights may represent dissimilarities between nodes, such as differences or distances. For instance, the weights in a power system network may stand for the distances between power stations. If two stations are far away from each other, the probability of the existence of a link between them is small. Under this situation, the weights are negatively correlated with the similarities of node pairs, and ulteriorly, negatively correlated with the connection likelihoods of links. In this case, if we directly apply such weights in Eqs (1)–(3) or (16)–(18) for link prediction, the results are worse than their unweighted cases accordingly, and this phenomenon is elucidated as the effects of weak ties on link prediction in Ref. [24]. Hence, in order to provide more accurate predictions, we should emphasize the role of weak ties as in Eqs (4)–(6) and (19)–(21). Consequently, the “revised weights” contribute positively to the connection likelihood. In USAir, the weight of a link represents the traffic flow between two airports. It’s indicated that the role of weak ties is more significant than strong ties in this network. Given that most airports are local ones and only a few are hubs connecting different local airports, if two local airports have higher frequent flights to the same hub airport, then the probability of direct flight between these two local airports are lower. In this way, the weights of USAir are negatively correlated with the connection likelihoods of links. Consequently, the role of weak ties in such a network is emphasized as indicated in Table 2.

Altogether, if the weights are positively correlated with the similarities of node pairs, the role of weak ties is depressed. Otherwise, the role of weak ties should be advocated.

Moreover, our model can also be used to predict the weights of missing links, which is also a significant task of link prediction in weighted networks. If the weights have a positive correlation with the connection likelihoods of links, we can use our model to get a score for each link, and then use the positive correlation between weights and scores to predict the missing weights (such as the method proposed in Ref [33]). On the contrary, if the weights denote the dissimilarities between nodes, the parameter *α* employed in Eqs (4)–(6) and (19)–(21) attempts to “modify” the weights to obtain a positive correlation with the similarities between nodes. After this modification, the method proposed in Ref [33] can then be applied to predict the “revised” weight, and finally the original weight can be predicted.

## Conclusion

In this paper, we propose a weighted mutual information model for link prediction in weighted networks, which combines the benefits from both structural properties and link weights. To test our method, empirical experiments are carried out on four real-world networks. The comparisons are made from two aspects. On the one hand, comparing to unweighted indices, without considering the fact of weak ties, the pure WMI-based indices can overwhelm their basic unweighted forms and achieve competitive performance with the LNB model in Celegans and Baywet. In addition, by taking the weak ties into consideration, the WMI model always performs the best in most networks. On the other hand, compared with other weighted indices, the WMI model also overwhelms them in most networks. Furthermore, experiments on four real-world networks demonstrate that the WMI model enjoys reasonable computing time. Altogether, we conclude that the WMI model is effective in link prediction of weighted networks.

The presented unweighted indices extract information from CN-based structures, and they perform well in high clustering networks, such as Bible. However, when the network has low clustering, these unweighted indices based on only structure information perform poorly. In this case, our model could handle this situation well by additional weight information of links. Although our model has some advantages over previous methods, it may cost more time to search for a reasonable parameter value when the role of weak ties needs to be addressed. Further investigation and improvements include but not limited to following aspects. The proposed model combines the weight information and structure information in a brief way. Therefore, more efficient ways need to be explored. In addition, since the weights of links may not show the real strength of ties, we may try to reconstruct a weighted network where original link weights are replaced by the values that estimate the tie strength more accurately, which will facilitate the weighted indices for capturing similarities between nodes.

## References

[1] Liben-Nowell D, Kleinberg J. The link-prediction problem for social networks. J Am Soc Inf Sci Technol. 2007;58(7):1019–1031. 10.1002/asi.20591

[2] Lü L, Zhou T. Link prediction in complex networks: A survay. Physica A. 2011;390(6):1150–1170. 10.1016/j.physa.2010.11.027

[3] Wang P, Xu BW, Wu YR, Zhou XY. Link prediction in social networks: the state-of-the-art. Sci China Inform Sci. 2015;58(1):1–38.

[4] Yu H, Braun P, Yıldırım MA, Lemmens I, Venkatesan K, Sahalie J, et al High-Quality Binary Protein Interaction Map of the Yeast Interactome Network. Science. 2008;322(5898):104–110. 10.1126/science.1158684 18719252 PMC2746753

[5] Jeong H, Tombor B, Albert R, Oltvai ZN, Barabási AL. The large-scale organization of metabolic networks. Nature. 2000;407(6804):651–654. 10.1038/35036627 11034217

[6] Guimerá R, Sales-Pardo M. Missing and spurious interactions and the reconstruction of complex networks. Proc Natl Acad Sci U S A. 2009;106(52):22073–22078. 10.1073/pnas.0908366106 20018705 PMC2799723

[7] Clauset A, Moore M, Newman MEJ. Hierarchical structure and the prediction of missing links in networks. Nature. 2008;453(7191):98–101. 10.1038/nature06830 18451861

[8] Resnick P, Varian HR. Recommender systems. Commun ACM. 1997;40(3):56–58. 10.1145/245108.245121

[9] Lü L, Medo M, Yeung CH, Zhang YC, Zhang ZK, Zhou T. Recommender systems. Phys Rep. 2012;519(1):1–49. 10.1016/j.physrep.2012.02.006

[10] Huang Z, Li X, Chen H. Link prediction approach to collaborative filtering. In: Proceedings of the 5th ACM/IEEE-CS joint conference on Digital libraries. ACM Press, New York; 2005. p. 141–142.

[11] Kleinberg J. Analysis of large-scale social and information networks. Phil Trans R Soc A. 2013;371(1987):20120378 10.1098/rsta.2012.0378 23419847

[12] Zhang QM, Lü L, Wang WQ, Zhu YX, Zhou T. Potential theory for directed networks. PLoS ONE. 2013;8:e55437 10.1371/journal.pone.0055437 23408979 PMC3569429

[13] Wang WQ, Zhang QM, Zhou T. Evaluating network models: A likelihood analysis. Europhys Lett. 2012;98(2):28004 10.1209/0295-5075/98/28004

[14] Zhang QM, Xu XK, Zhu YX, Zhou T. Measuring multiple evolution mechanisms of complex networks. Scientific Reports. 2015;5(10350). 10.1038/srep10350 PMC4464182 26065382

[15] Lü L, Pan L, Zhou T, Zhang YC, Stanley HE. Toward link predictability of complex networks. Proc Natl Acad Sci U S A. 2015;112(8):2325–2330. 10.1073/pnas.1424644112 25659742 PMC4345601

[16] Adamic LA, Adar E. Friends and neighbors on the web. Social networks. 2003;25(3):211–230. 10.1016/S0378-8733(03)00009-1

[17] Zhou T, Lü L, Zhang YC. Predicting missing links via local information. Eur Phys J B. 2009;71(4):623–630. 10.1140/epjb/e2009-00335-8

[18] Liu Z, Zhang QM, Lü L, Zhou T. Link prediction in complex networks: A local naïve Bayes model. Europhys Lett. 2011;96(4):48007 10.1209/0295-5075/96/48007

[19] Liu H, Hu Z, Haddadi H, Tian H. Hidden link prediction based on node centrality and weak ties. Europhys Lett. 2013;101(1):18004.

[20] Tan F, Xia Y, Zhu B. Link Prediction in Complex Networks: A Mutual Information Perspective. PLoS ONE. 2014;9(9):e107056 10.1371/journal.pone.0107056 25207920 PMC4160214

[21] Murata T, Moriyasu S. Link prediction of social networks based on weighted proximity measures. In: Proceedings of the IEEE/WIC/ACM international conference on Web Intelligence. ACM Press, New York; 2007. p. 85–88.

[22] Granovetter MS. The strength of weak ties. Am J Sociol. 1973;78:1360–1380. 10.1086/225469

[23] Csermely P. Weak links: Stabilizers of complex systems from proteins to social networks. Springer-Verlag, Berlin/Heidelberg; 2006.

[24] Lü L, Zhou T. Link prediction in weighted networks: The role of weak ties. Europhys Lett. 2010;89(1):18001 10.1209/0295-5075/89/18001

[25] Sá HR, Prudêncio RB. Supervised learning for link prediction in weighted networks. In: Proceedings of the III International Workshop on Web and Text Intelligence; 2010.

[26] Watts DJ, Strogatz SH. Collective dynamics of’small-world’ networks. Nature. 1998;393(6684):440–442. 10.1038/30918 9623998

[27] Pajek datasets; 2006. Available: http://vlado.fmf.uni-lj.si/pub/networks/data/

[28] The Koblenz Network Collection; 2015. Available: http://konect.uni-koblenz.de/

[29] Shannon CE. A mathematical theory of communication. ACM SIGMOBILE Mobile Computing and Communications Review. 2001;5(1):3–55. 10.1145/584091.584093

[30] Cover TM, Thomas JA. Elements of information theory. John Wiley & Sons; 2012.

[31] Du WB, Gao Y, Liu C, Zheng Z, Wang Z. Adequate is better: particle swarm optimization with limited-information. Applied Mathematics and Computation. 2015;268:832–838. 10.1016/j.amc.2015.06.062

[32] Zhu B, Xia Y. An information-theoretic model for link prediction in complex networks. Scientific Reports. 2015;5(13703). 10.1038/srep13707 PMC4558573 26335758

[33] Zhao J, Miao L, Yang J, Fang H, Zhang QM, Nie M, et al Prediction of Links and Weights in Networks by Reliable Routes. Scientific Reports. 2015;5(12261). 10.1038/srep12261 PMC4510530 26198206

